# APOC-III: a Gatekeeper in Controlling Triglyceride Metabolism

**DOI:** 10.1007/s11883-023-01080-8

**Published:** 2023-01-23

**Authors:** Antonina Giammanco, Rossella Spina, Angelo B. Cefalù, Maurizio Averna

**Affiliations:** 1 Department of Health Promotion, Mother and Child Care, Internal Medicine and Medical Specialties “G. D’Alessandro” (PROMISE), University of Palermo, Palermo, Italy; 2 Institute of Biophysics (IBF), National Research Council (CNR), Palermo, Italy

**Keywords:** ApoC-III, Triglyceride-rich lipoproteins (TRLs), Cardiovascular disease burden, Post prandial lipemia, Therapeutic target

## Abstract

**Purpose of Review:**

Apolipoprotein C-III (ApoC-III) is a widely known player in triglyceride metabolism, and it has been recently recognized as a polyhedric factor which may regulate several pathways beyond lipid metabolism by influencing cardiovascular, metabolic, and neurological disease risk. This review summarizes the different functions of ApoC-III and underlines the recent findings related to its multifaceted pathophysiological role.

**Recent Findings:**

The role of ApoC-III has been implicated in HDL metabolism and in the development of atherosclerosis, inflammation, and ER stress in endothelial cells. ApoC-III has been recently considered an important player in insulin resistance mechanisms, lipodystrophy, diabetic dyslipidemia, and postprandial hypertriglyceridemia (PPT). The emerging evidence of the involvement of ApoC-III in the in the pathogenesis of Alzheimer’s disease open the way to further study if modification of ApoC-III level slows disease progression. Furthermore, ApoC-III is clearly linked to cardiovascular disease (CVD) risk, and progression of coronary artery disease (CAD) as well as the calcification of aortic valve and recent clinical trials has pointed out the inhibition of ApoC-III as a promising approach to manage hypertriglyceridemia and prevent CVD.

**Summary:**

Several evidences highlight the role of ApoC-III not only in triglyceride metabolism but also in several cardio-metabolic pathways. Results from recent clinical trials underline that the inhibition of ApoC-III is a promising therapeutical strategy for the management of severe hypertriglyceridemia and in CVD prevention.

## Introduction

Apolipoprotein C-III (ApoC-III) is a small apoprotein (79 amino acid residues) that mainly resides in triglyceride-rich lipoproteins (TRLs) (chylomicrons, very low density lipoproteins (VLDL), and their remnants), low density lipoproteins (LDL), and high density lipoproteins (HDL). Current knowledge on ApoC-III suggests a multifaceted influence in several pathophysiological processes including TRLs metabolism, atherosclerosis, inflammations, glucose metabolism, and cardiovascular and neurological diseases [1].

## ApoC-III: Structure, Genetic, and Glycoform Distribution in Plasma

*APOC3* gene maps on chromosome 11q23 closely linked with *APOA1* and *APOA4* genes in a cluster on 17 kb *APOA1/C3/A4/A5* gene cluster [2]. ApoC-III, first described in 1969 by Brown, is a glycoprotein synthesized in intestine and liver and consists of 99-aa residues. The mature form lacks the first 20-aa residues of signal peptide [3]. Different glycoforms of ApoC-III have been characterized, and each of them influences TG metabolism [4]. These post-translational modifications of ApoC-III are distinguished by different levels of sialic acid residues (native forms, mono-sialic acid, bi-sialic acid), and each form plays a distinctive role in lipid metabolism. ApoC-III is a high interchangeable protein, and it can be detected in all different types of lipoproteins (VLDL, intermediate density lipoproteins — IDL, LDL, HDL) [4]. There is a significant inter-individual variability on distribution of ApoC-III glycoforms in lipoproteins, but their proportion across lipoprotein fractions is maintained [4]. However, the presence or absence of ApoC-III in specific lipoproteins may contribute to atherosclerotic cardiovascular disease (CVD) [5].

## ApoC-III and TRL Metabolism

ApoC-III is an important apolipoprotein involved in TRL metabolism as a cofactor of lipoprotein lipase (LPL). LPL is a central regulator of lipid metabolism and plays a key role in the hydrolysis of VLDL and chylomicrons. ApoC-III is a well-known inhibitor of LPL and hampers the hepatic uptake of TG-rich lipoproteins by an independent LPL pathway [6].

ApoC-III serum concentration widely differ between normolipidemic subjects (~ 8–10 mg/dl) and hypertriglyceridemic subjects (HTG) (24–30 mg/dl) [5, 7].

ApoC-III inhibits LPL-mediated lipolysis, acts on the ApoE-mediated hepatic uptake of VLDL particles, promotes the assembling and secretion of VLDL [8, 9], and suppresses the TRL remnant clearance in the liver. The kinetics of ApoC-III in plasma determine the clearance of TRL through interactions with ApoE [5, 7]. In normotriglyceridemic subjects, the majority of TRL is secreted together with ApoE and determines a rapid clearance from the blood stream through ApoE-dependent uptake mechanisms in the liver. On the contrary, in patients with hypertriglyceridemia, TRL metabolism changes from an apoE-dominated manner to an ApoC-III–dominated system, which contributes to decreased VLDL clearance [5, 7].

It is not still clear how ApoC-III inhibits LPL, and different studies have proposed different hypotheses. Hypertriglyceridemic *APOC3* transgenic mice have shown that ApoC-III may inhibit either the ApoE-mediated hepatic uptake of TRLs or the hydrolysis of TG LPL-mediated. More, hypotriglyceridemic *APOC3* knockout (*apoC3*(-/-)) mice intercrossed with apoE (-/-) hyperlipidemic mice display low levels of VLDL cholesterol and TG similar to apoE (+ / +) apoC3 (-/-) mice indicating that the mechanisms by which ApoC-III deficiency induces its lipid-lowering effect are independent of ApoE [9]. These studies help to understand that ApoC-III is an effective inhibitor of VLDL-TG hydrolysis and highlights a potential role for ApoC-III in regulating the selective uptake of cholesteryl esters [9].

Early studies testing antisense oligonucleotide (ASO) inhibition of ApoC-III have shown in preclinical animal models and in a phase I clinical study in healthy subjects a consistent reduction of both ApoC-III and TG in a dose-dependent manner [10]. In addition, Gordts et al. demonstrated that ApoC-III antisense inhibition in mice prevents TRL clearance via both low-density lipoprotein receptors (LDLRs) and LDLR-related protein 1 (LRP1) receptors, thus indicating that ApoC-III inhibits TRLs turnover mostly through a hepatic clearance mechanism [8].

## ApoC-III and HDL Metabolism

ApoC-III is transferred between VLDL and two main subfractions of HDL, HDL2 and HDL3 [11]. Although ApoC-III is transferred bidirectionally between VLDL and HDL, experimental data indicates that ApoC-III is dominantly transferred from VLDL to HDL. In the blood stream, the particle size of HDL3 becomes larger due to the esterification of surface free cholesterol by lecithin-cholesterol acyltransferase (LCAT) and the exchange of TG and esterified cholesterol between VLDL and HDL by CETP; the larger HDL3 eventually converts to HDL2. CETP is not necessarily involved in apolipoprotein transfers and variable levels of ApoC-II/ApoC-III seen among healthy subjects maybe due to variability in TG metabolism among individuals [11].

## ApoC-III Genetic Variation, Serum TG, Risk of Coronary Artery Disease, and Cardiovascular Disease Burden

Epidemiological and observational studies have demonstrated that ApoC-III elevated plasma levels correlate with higher TG and higher risk of ASCVD and progression of coronary artery disease (CAD) [12–14]. However, ApoC-III atherogenic lipid particles (ApoC-III-apoB, apoC-III-Lp(a), and apoC-III-apoA-I) do not independently predict risk of CAD events as total ApoC-III do [15]. The first evidence of the relationship between ApoC-III and ASCVD risk comes from a study by Pollin et al. [16] which demonstrated that carriers of a rare *APOC3* null-mutation have lower TG levels and a lower coronary calcium score. These data were later on confirmed by two milestone studies which correlated loss of function (LOF) mutations of *APOC3* to a 40% ASCVD risk decrease [17, 18]. A similar association was seen among 2707 participants from the Framingham Heart Study: an *APOC3* mutation was associated with a 46% lower plasma Apo-CIII level, and a 1 mg/dl reduction of ApoC-III level was associated with a 4% lower risk of incident ASCVD [18, 19].

Loss of function mutations of *APOC3* also affect levels of non-fasting TG and risk of atherosclerotic cardiovascular disease. In fact, the loss-of-function mutations (R19X, IVS2 + 1G > A, and A43T) in the *APOC3* gene, identified in 75,725 participants belonging to two general-population, are associated with lower circulating TG and remnant cholesterol levels (44% reduction) and with a lower burden of coronary artery calcification, which represents a surrogate marker for atherosclerosis [16]*.* In carriers of LOF mutations of *APOC3*, the cardio-protective lipid profiles is also characterized by significantly higher levels of HDL-C and lower total cholesterol levels compared with non-carriers [20, 21].

The finding that LOF carriers of *APOC3* gene mutations exhibit a favorable lipid profile and lower risk of ASCVD have been confirmed in several studies including multiple racial/ethnic groups [22]. Pollin et al. [16] showed that approximately 5% of Amish subjects carrying the R19X null allele of the *APOC3* gene exhibit a favorable lipid profile and a low extent of coronary artery calcification. The TG and HDL Working Group of the Exome Sequencing Project identified in a cohort of 3734 subjects several rare coding variants of *APOC3* gene associated with a large effect on plasma TG levels [23]. The link between *APOC3* loss-of-function mutations and low risk of ischemic vascular disease (IVD) was also confirmed in a meta-analysis by Wulff AB et al. [24] in which it was reported that carriers of *APOC3* loss-of-function heterozygotes exhibit lower remnant cholesterol levels and, respectively, 37% and 54% risk reduction of IVD and ischemic heart disease (IHD).

Kathiresan et al. identified four rare variants in *APOC3* that were associated with a 39% decrease in plasma TG levels [23]. He sequenced the exons from 18,666 genes from 3734 participants in seven population-based cohorts of European and African ancestry, who had measurable fasting plasma TG levels. The variants were then tested for association with CHD in 110,097 individuals from 15 different studies [25]: it has been descripted that rare LOF *APOC3* mutations decrease both TG and ApoC-III plasma levels and in the same time reduce the CHD risk, since mutation carriers exhibited a 40% decrease in CHD compared to non-carriers. More, in a cohort of 75,725 Danes, carriers of these variants had a 41% reduction in CHD. Silbernagel et al. [26] studied seven common variants in *APOC3* (rs734104, rs4520, rs5142, rs5141, rs5130, rs5128, and rs4225) associated with high levels of APOCIII, TG, VLDL, and TC, but no elevations of LDL and ApoB levels were observed. All these variants do not correlate with CAD. Recently, Goyal et al. [27] sequenced 396,644 individuals from India, Europe, Mexico, and Singapore and identified two variants, rs5128, mapped on 3′ UTR of ApoC-III, and rs734104, an intronic variant. These two variants correlated with a significant increase of TG, while no association was observed with T2D, LDL cholesterol, and total cholesterol. They investigated about null and rare *APOC3* variants and the correlation with TG levels and CAD; some variants have shown different effects in different patients, and even though the plasma TG levels mean was significantly lower among the carriers of the variant allele vs. wild type, none of these variants exhibited any significant protection against CAD except for the rs138326449 [27].

A recent meta-analysis reported the association between two *APOC3* gene polymorphisms (SstI and T-455C) and increased risk of coronary heart disease (CHD) [28].

Overall, these findings strongly suggest that ApoC-III is an important pharmacological target for managing dyslipidemia and CVD risk [13].

## ApoC-III, Postprandial Lipemia, and Atherosclerosis

Plasma LDL cholesterol reduction is a key goal for atherosclerotic cardiovascular disease (ASCVD) prevention [29], but apolipoproteins (Apos), such ApoB and ApoCs, highly contribute as casual factor, because it quantitatively measures the number of LDL and of VLDL and remnants [30–32]*.* VLDL, as compared to LDL, is enriched with TG that significantly increases in non-fasting status and even more in the so-called postprandial lipemia (PPL) [33]. PPL was originally considered as an elevation of TG following consumption of high fat-based meals (20–40 g/fats) that are consumed daily in nowadays affluent societies [32]. PPL has been also recently described to last by 6–8 h [34], hence being a daily and iterative situation accumulating over time. The evidence on the importance of PPL is rising as it has been clearly recognized as being associated with elevated CVD [35]. Epidemiological data and Mendelian randomization approaches support the elevated atherogenic potential of VLDL in the non-fasting status [36•, 37] and experimental evidence underscore inflammatory effects of these particles occurring during PPL [38]. Whether these effects are mediated by cholesterol per se [39] or are consequences of other lipids contained in VLDL is hard to dissect. To what extent the lipid content in VLDL, in LDL, or in both promotes these key long-lasting mechanisms is still debated and has an immediate clinical relevance. ApoC-III, as previously reported, is a key factor in TG metabolism [40, 41]; it inhibits lipoprotein lipase (LPL) and influences the uptake of TRLs from the liver [42]. It also influences the activity of hepatic lipase (HL), which is involved in the conversion of VLDL to intermediate density lipoprotein (IDL) and LDL. In vivo studies performed in mice and humans have reported that ApoC-III plasma levels are increased by post prandial (PP) free fatty acids [43], and recently, Guan Y et al. have performed a study to investigate possible correlations between ApoC-III and PPL [44]: they have found that subjects with PP hypertriglyceridemia (PPT) have both fasting and PP elevated levels of ApoC-III, which represents an independent risk factor of PPT as well as a causative factor and may be considered a possible biomarker of PPT itself. In this context, controlling the ApoC-III plasma levels could be an effective strategy to ameliorate PPT and in the same time prevent atherosclerosis and cardiovascular diseases [44]. Furthermore, chylomicron-free serum ApoC-III levels both in fasting and PP state may represent a valuable predictor of recurrent cardiovascular events in patients with stable CAD [45••].

ApoC-III is associated to atherosclerosis and coronary heart disease (CHD) due to its proatherogenic properties, the inhibition of the catabolism, and the clearance of TLRs which induce hypertriglyceridemia [46]. ApoC-III-rich VLDL induce the monocyte adhesion to endothelial cells and the subsequent activation of proinflammatory mechanisms that determine atherogenesis progression. Different genetic studies have reported a causal role of ApoC-III and CAD risk as well as it has been demonstrated that loss-of-function mutations of *APOC3* decrease CAD risk [18, 47, 48]. Lipoprotein-associated ApoC-III [apoB, apoAI, Lp(a)] levels have been associated to coronary artery disease (CAD) risk in the EPIC-Norfolk prospective population study including 25,663 subjects [15]. In this study, it was evaluated the distribution of ApoC-III among lipoproteins, and it was found that lipoprotein-associated ApoC-III reflect atherogenic lipids but, on the contrary of total plasma ApoC-III measurement, are not predictive of CAD occurrence.

ApoC-III interacts also with apoE in modulating HDL metabolism and CAD risk [48], and four large prospective cohort studies have supported the idea that HDL-rich in ApoC-III correlate with increased CHD risk [49]. Based on this finding, the absence of ApoC-III is associated to lower CHD risk. The effect of ApoC-III is mostly due to its role in HDL apoA-I clearance as it has been demonstrated by lowering plasma ApoC-III levels with fibrates and statins [50]. To identify predictor factors of severity in CAD, recently Braiek AB et al. have studied a panel of biomarkers involved in atherosclerotic plaque instability and rupture including matrix metalloproteinases (MMPs) and specific tissue inhibitors (TIMPs) which controls MMP functions [51], and they conclude that ApoC-III as well as ApoC-II positively correlates with MMPs and negatively with TIMPs, thus suggesting a synergic performance of these factors in cardiovascular disorders.

## ApoC-III and Inflammation, Atherosclerosis, and Endoplasmic reticulum (ER) Stress

Atherosclerosis begins with vascular endothelial dysfunction, activation and recruitment of monocytes to the vascular wall, differentiation into macrophages, uptake of cholesterol, and other lipoproteins and formation of foam cells. Oxidate LDL particles have a crucial role in the development of atherosclerosis, but recently, new particles have been found to be involved in this process. In fact, TRL particles were found to participate to leukocyte activation, endothelial dysfunction, and the formation of foam cells [52, 53]. Li H et al. [54] have shown that ApoC-III directly promotes the expression and activation of the vascular cell adhesion molecule-1 (VCAM-1) in human umbilical vein endothelial cells (HUVECs) and induces monocyte adhesion with an increase of inflammatory response and an important contribute to atherosclerosis progression. ApoC-III shows proinflammatory properties related to its ability to increase binding to proteoglycans and monocytes to cultured endothelial cells through the induction of VCAM-1: for this reason, it has an important potential to facilitate atherosclerosis [18]. In a recent study, Yingchun et al. [55] investigated about the role of ApoC-III and triglycerides in the development of inflammation and ER stress levels in endothelial cells. They conducted studies on ApoC-IIItg/LDLR − / − and LDLR − / − and HUVEC cells and demonstrated that ApoC-III increases atherosclerotic lesions and promotes the expression of VCAM-1 and monocyte chemoattractant protein-1 (MCP-1) and expression of oxidative stress and ER stress-related proteins. They also studied TRLs ± ApoC-III that markedly increased the protein expression of protein disulfide isomerase (PDI), but there was no significant difference between the two types of TRLs suggesting that ER stress in macrophages was induced by TRLs independently of ApoC-III.

## Apo-CIII: Diabetes Mellitus and Insulin Resistance

ApoC-III has been recently considered an important player in the insulin resistance mechanisms and the development of dyslipidemia in type 2 diabetes (T2DM) [56]: ApoC-III levels were linked to pancreatic islet insulin resistance and β-cell dysfunction [56] mostly due to the progressive inflammation and induction of β-cell apoptosis and decline. ApoC-III production is stimulated by insulin resistance, but ApoC-III itself amplifies insulin resistance through endothelial cells (ECs) [46] by contributing to an impaired insulin signaling and endothelial dysfunction, acting as a key driver of the diabetic dyslipidemia [46]. The endothelial dysfunction is characterized by a decrease of nitric oxide (NO) and its vasodilatory and anti-atherogenic properties. Insulin induces endothelial NO synthase (eNOS) in ECs and determines the production of NO; insulin resistance, consequently, causes ECs dysfunction. ApoC-III activates protein kinase C beta (PKC β) which inhibits insulin signaling in ECs through eNOS pathway and the production of NO in ECs, thus determining endothelial dysfunction [51]. The pro-diabetogenic role of ApoC-III was demonstrated also by Qamar et al. which found a positive correlation between ApoC-III levels and fasting glucose/glycosylated hemoglobin A1C (HbA1c) in T2DM patients [57]. Furthermore, ApoC-III plasma levels correlated with hypertriglyceridemia and increased coronary artery calcification in dyslipidemic subjects with T2DM [57].

*APOC3* gene expression is suppressed by insulin through the nuclear transcription factor Foxo1 which regulate the activity of the apoC3 promoter [58]; in contrast to insulin, *APOC3* gene expression is induced by glucose through a mechanism still unknown, thus contributing to diabetic dyslipidemia [59]. Recently, Sigfrids FJ et al. have performed a study including 3966 subjects with type 1 diabetes to evaluate a predictive role of ApoC-III in diabetic kidney disease (DKD), major adverse cardiac events (MACE), and mortality: it was reported a positive correlation between ApoC-III and cardiovascular events in type 1 diabetic presenting with albuminuria, thus predicting DKD progression and mortality [60•]. An important correlation between enhanced insulin sensitivity and both plasma ApoC-III and TG suppression was also reported through the effects of the antisense *APOC3* inhibitor — volanesorsen (ISIS 304801) — on TG levels and insulin resistance in patients with type 2 diabetes [61].

## ApoC-III: Obesity and Lipodystrophy

In agreement to in vivo studies performed on both transgenic mice overexpressing ApoC-III and knock-out mice with lacking ApoC-III, it has been shown that this apolipoprotein regulates the metabolic mechanisms underlying diet-induced obesity in the liver and in the brown adipose tissue [62–64]. In particular, decreasing ApoC-III ameliorates insulin sensitivity and triglyceride levels [65]. In a very recent study focused on subcutaneous (SAT) and visceral (VAT) white adipose tissue (WAT), Recio-Lopez et al. have found that lowering ApoC-III determines improvements of metabolic status, decreases inflammation and size of adipocytes in WAT, and enhances the expression of genes related to thermogenesis and functions of SAT [66].

Familial partial lipodystrophies (FPLD) are rare genetic disorders characterized by marked loss of subcutaneous fat from the extremities with variable fat loss from the face and trunk. Patients with FPLD develop metabolic abnormalities including hypertriglyceridemia, insulin resistance, and diabetes mellitus, which are difficult to manage with conventional therapies including fibrates, statins, and insulin [67].

Hypertriglyceridemia is a complication of lipodystrophy mainly due to the effect of leptin deficiency and de novo lipogenesis [68, 69]: as over insulin-resistant state, it is secondary to an inefficient capacity to store surplus energy, elevated free fatty acid turnover, VLDL secretion, and decreased LPL activity [68]. Leptin replacement in patients with lipodystrophy reduces serum TGs [68].

ApoC-III plays a role in the pathogenesis of insulin-resistance and hypertriglyceridemia observed in lipodystrophy: ApoC-III levels are higher in lipodystrophy patients compared with overweight/obese controls independently of leptin deficiency and replacement [68].

ApoC-III and other LPL modulators, such ANGPTL3 and ANGPTL8, decrease significantly after the administration of metreleptin, a recombinant human methionyl leptin, thus leading to triglyceride lowering and liver fat reduction [70]**.**

Recent clinical trials support the role of *APOC3* inhibition in lipodistrophy. Two studies — NCT02639286 (data unpublished yet) and NCT02527343 [71] — have supported the role of antisense oligonucleotide anti-*APOC3* in patients with partial lipodystrophy. Leptin replacement may decrease ApoC-III by reducing plasma fasting glucose levels, as it has been demonstrated in leptin deficient ob/ob mice [68], but in humans, it is not enough to manage the metabolic complications of lipodystrophy, in particular hypertriglyceridemia [70]*.* For this reason, ApoC-III inhibitors may represent a valuable therapeutic option in the future for treatment of lipodystrophy.

## ApoC-III and Alzheimer’s Disease

ApoC-III as well as the other members of the apolipoprotein C family (ApoC-I and ApoC-II) interact with ApoE and influence the pathophysiology of Alzheimer’s disease (AD) [72]. AD is a progressive neurodegenerative disorder that causes irreversible brain atrophy and the accumulation of senile plaque and amyloid β, representing the most common cause of dementia [72]. Recently Chan HC et al. have demonstrated that ApoC-III-rich HDL particles are associated with AD [73]. ApoC-III co-localize with ganglioside GM1 alongside an increased tumor necrosis factor-α (TNF-α) levels; therefore, it has been supposed a potential proinflammatory role of ApoC-III involved in the blood–brain barrier destruction and amyloid β deposition in AD patients [73]*.* Another interesting finding is the correlation between ApoC-III plasma isoforms and the cerebrospinal fluid (CSF): the ApoC-III delivery takes place through the apoA-I containing HDL, which makes this transport possible due to their typical discoidal shape [72]. Since ApoC-III levels are increased in CSF of AD’s patients, it has been hypothesized that ApoC-III on TRLs may promote the efflux of Amyloid β from the brain [73, 74], thus influencing the AD mechanisms of Aβ assembly and neural inflammation.

## ApoC-III and Calcification of Aortic Valve

ApoC-III has been implicated in aortic valve calcification [75] through a mechanism inducing mitochondrial dysfunction and oxidative stress of primary human valvular interstitial cells (VICs) [76]. These cells under specific triggers, i.e., inflammation, are subjected to differentiate in myofibroblasts, thus supporting the formation of fibrous tissue and calcification determining calcific aortic valve disease (CAVD) and aortic stenosis (AS) [76]. ApoC-III is increased both in human calcific CAVD tissue and around calcific nodules: this finding has risen the idea that it is directly involved in the promotion of AV calcification beside its other metabolic functions. The IL-6/BMP-2 pathway is involved in the VIC calcification process as in vitro studies have been shown [75]. Furthermore, ApoC-III enhances proinflammatory signaling pathways [77, 78] determining calcification and the activation of CAVD-specific factors cascade [79].

**Fig. 1:**
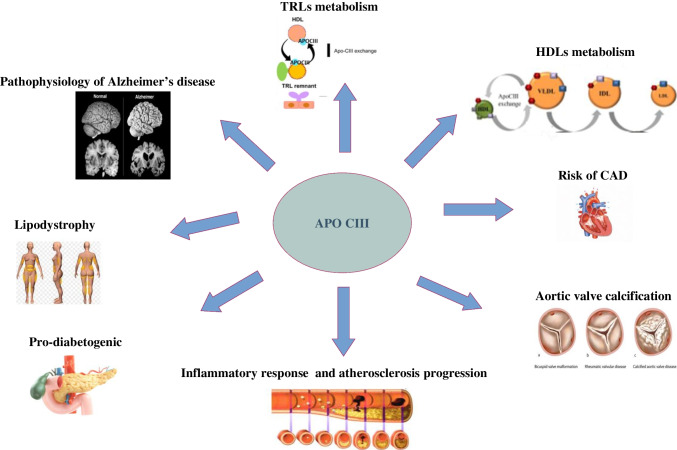
Schematic representation of ApoC-III roles and functions discussed in this review. ApoC-III prevents TRL clearance via both low-density lipoprotein receptors (LDLRs) and LDLR-related protein; bidirectional transfer between VLDL and HDL in a CETP-independent manner; it inhibits LPL and obstructs the hepatic uptake of TG-rich lipoproteins, enhances proinflammatory signaling pathways (IL-6/BMP-2), promotes the expression of VCAM-1 and MCP-1 and expression of oxidative stress and ER stress-related proteins, and correlates to diabetes mellitus and insulin-resistance; obesity amplifies insulin resistance through endothelial cells; it is involved in the development of hypertriglyceridemia observed in lipodystrophy, increases tumor necrosis factor-α (TNF-α) levels, and may promote the efflux of Amyloid β from the brain

Figure 1 represents schematically the ApoC-III role and functions reported in this review.

## ApoC-III Inhibition as Novel Therapeutic Strategy

ApoC-III inhibition by antisense oligonucleotides (ASO) represents so far a unique and effective strategy to lower ApoC-III levels [80]. Monoclonal antibodies anti-ApoC-III, STT505, and STT5058, appear to decrease ApoC-III levels and determine TRLs clearance in animal models [81]. Gaudet D et al. reported firstly the antisense inhibition with volanesorsen administered subcutaneously in hypertriglyceridemic patients demonstrating an important TG decrease (from –40 to − 79.6%) [82]; even on 66 patients with familial chylomicronemia syndrome (FCS) treated for 52 weeks with this ASO, the TG reduction was significant (− 77%) as well as ApoC-III levels lowering (− 84%). Nevertheless, despite volanesorsen determined adverse events including severe thrombocytopenia, it was approved and authorized by the European Medical Agency (EMA) which evaluated favorably the benefit: risk ratio [83•]. A recent meta-analysis of the phase 2 and phase 3 clinical trials on volanesorsen underlined the efficacy of volanesorsen in lowering VLDL-C (− 73%), TG (− 68%), ApoC-III (− 74%), and increasing HDL-C (+ 40%) and LDL-C (+ 47%) [83•]. Recently, a newly ASO anti-ApoC-III is under phase 1/2a study: it is a modified ASO, N-acetyl galactosamine (GalNac3)-conjugated APOCIII-L_Rx_ which targets the asialoglycoprotein receptor (ASGPR) in the hepatocytes and seems effective in TG and ApoC-III decrease without inducing any significant adverse events such low platelet count, flu-like symptoms, and skin injection site reactions [84, 85]. Further studies are needed to evaluate the efficacy and safety of ApoC-III inhibition both in TG lowering and in CVD prevention. To date, the ongoing trial ISIS 678354 (AKCEA-APOCIII-LRx) in patients with hypertriglyceridemia and established CVD might unravel and elucidate this point [ClinicalTrials.gov/ct2/show/NCT03385239].

## Conclusion

Several evidences highlight the role of ApoC-III not only in triglyceride metabolism but also in several cardio-metabolic pathways. Clinical trials have underlined that ApoC-III inhibition is an effective strategy for the management of severe hypertriglyceridemia. In the future, novel therapies directed toward ApoC-III inactivation may offer promising therapeutical strategy to manage severe hypertriglyceridemia and CVD prevention.
